# Supplementary material for: Crosslinking of fibrous hydrogels
Source: Nat Commun. 2018 Jun 4;9:2172. doi: 10.1038/s41467-018-04508-x (PMC5986759; doi:10.1038/s41467-018-04508-x)
Supplement: Supplementary file 1 — Supplementary Information [file 41467_2018_4508_MOESM1_ESM.pdf]

Supplementary Methods

Synthetic procedures

General. ^{1}H- and ^{13}C-NMR spectra were recorded on a *Bruker Avance III 500MHz* (126 MHz for ^{13}C) spectrometer. All MALDI-TOF spectra were recorded on a *Bruker Microflex LRF MALDI-TOF* system using dithranol as matrix. For viscometry measurements the polymers were dissolved in acetonitrile (0.1 –0.6 mg ml^{-1}) and the intrinsic viscosity of these solutions was measured at T = 25 °C. The average viscosity molecular weight, M_v, of the polymers was calculated using the empirical Mark–Houwink equation: $[\eta] = KM_v^a$. Where $[\eta]$ is the intrinsic viscosity of the polymer solution as determined from viscometry measurements and Mark–Houwink parameters K and a depend on solvent and polymer characteristics. We used parameter values that were previously determined for other polyisocyanides[1]: $K = 1.4 \times 10^{-9}$ and $a = 1.75$. All compounds were used as purchased unless specifically mentioned otherwise.

Azide-functionalized isocyanide monomer. The azide-functionalized isocyanide monomer was synthesized following a modified literature procedure.[2] The first and last step of the procedure were altered.

Tetra(ethylene glycol) p-toluenesulfonate. Tetraethylene glycol (52.68 g, 271 mmol) was dissolved in 10 mL THF and the solution was cooled to 0 °C. A solution of NaOH (1.81 g, 45.25 mmol) in 10 mL water was added and the mixture was stirred vigorously for 5 minutes. A solution of tosyl chloride (8.08 g, 42.4 mmol) in THF (70 mL) was added drop wise. The reaction mixture was stirred for 2.5h at room temperature. Subsequently, the solution was poured onto ice water (200 mL) and DCM (50 mL) was added and the layers were separated. The aqueous layer was extracted 4 times with DCM (100 mL). The combined organic layers were dried using anhydrous $NaSO_4$ and the solvents were removed *in vacuo*. The product was purified using flash chromatography (SiO_2, EtOAc) to yield 11.9 g (80%) of a yellow oil. The analysis results matched those reported in literature.[2]

(R)-2-(2-(2-(2-azidoethoxy)ethoxy)ethoxy)ethyl 2-((S)-2-isocyanopropanamido)propanoate. (*R*)-2-(2-(2-(2-azidoethoxy)ethoxy)ethoxy)ethyl 2-((*S*)-2-formamidopropanamido)propanoate (640 mg, 1.64 mmol) was dissolved in freshly distilled DCM (60 mL). To this solution, methyl *N*-(triethylammoniumsulfonyl) carbamate (Burgess reagent; 594 mg, 2.49 mmol) was added. The mixture was stirred at room temperature for 6 h until all starting material was consumed (observed by TLC). The solvents were removed *in vacuo*, and the product was purified using flash chromatography (SiO_2, 3:1 DCM:MeCN) to yield 0.436 g (72%) of a pale yellow oil. The analysis results matched those reported in literature.[2]

Polyisocyanide polymer 1. The commercially available methoxy-terminated isocyanide monomer was purified by flash chromatography (SiO_2, 1:3 MeCN:DCM) before use. The purified monomer (250 mg, 0.79 mmol) and the azide functionalized isocyanide monomer (10 mg, 27 μmol) were dissolved in freshly distilled toluene (4 mL). A catalyst stock solution of 1 mM $Ni(ClO_4)_2{\cdot}6H_2O$ in freshly distilled toluene/ethanol (9:1) was prepared. 81.7 μL of the stock solution was diluted to 1 mL using freshly distilled toluene and added to the monomer solution. The resulting mixture was stirred for 72 h at room temperature. Afterwards, the reaction mixture was diluted with DCM (5 mL) and the product was precipitated in diisopropyl ether (100 mL). After filtration, the polymer was redissolved in DCM (5 mL) and reprecipitated in diisopropyl ether (100 mL). This procedure was repeated two more times. The product was obtained as a pale yellow solid, yield: 244 mg (94 %). FT-IR ν_{max} film (cm^{-1}): 3268, 2876, 1742, 1657, 1532, 1455, 1264, 1217, 1065, 729, 703. M_v = 599 kDa, UV-Vis λ_{max} (25 °C, milliQ) = 245 nm.

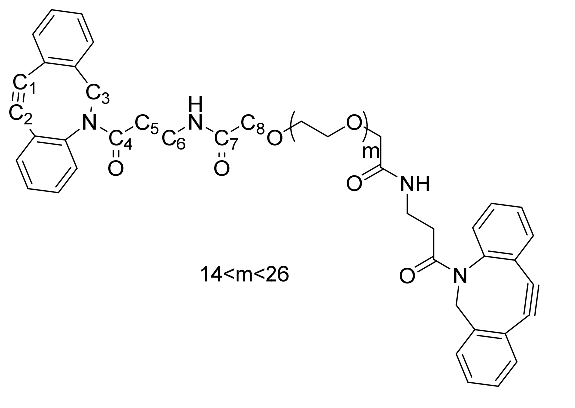

Bis-DBCO PEG(1000) crosslinker 2b. 4-Dimethylamino-pyridine (DMAP, 0.5 mg, 4.4 μmol), DBCO-amine (38.1 mg, 138 μmol) and PEG(1000) bis-acetic acid (50 mg, 44 μmol) were dissolved in freshly distilled DCM (5 mL). The reaction mixture was cooled to 0°C. 1-Ethyl-3-(3-dimethylaminopropyl)carbodiimide hydrochloride (EDC·HCl, 28 mg, 146 *μ*mol) was added and the mixture was allowed to warm up to room temperature. The solution was stirred overnight at room temperature. Afterwards, the reaction mixture was diluted with DCM and the organic layer was washed with water (3x50 mL) and brine (1x50mL). The organic layer was dried with anhydrous Na_2SO_4 and the solvents were evaporated *in vacuo*. The product was dissolved in THF (1 mL), filtered and the solvent was evaporated *in vacuo.* This procedure was repeated once. For further purification, the product was dissolved in a minimum amount of THF and precipitated in cold heptane. This procedure was repeated twice, after which the product was obtained as yellow oil with a yield of 59.7 mg (84 %). Analysis: ^{1}H-NMR (500 MHz, $CDCl_3$, ppm) δ: 7.70 (d, 2H, $C_{Ar}\underline{H}$), 7.46 – 7.31 (m, 14H, $C_{Ar}\underline{H}$), 7.13 (br t, 2H, N$\underline{H}$), 5.16 (d, 2H, $C_3\underline{H}$), 3.90 (q, 4H, $C_8\underline{H_2}$), 3.80 – 3.60 (m, 88H, -O-C$\underline{H_2}$-C$\underline{H_2}$-, $C_3\underline{H}$), 3.60 – 3.49 (m, 8H, -O-C$\underline{H_2}$-C$\underline{H_2}$-), 3.44 – 3.32 (m, 4H, $C_6\underline{H_2}$), 2.55 (dt, 2H, $C_5\underline{H}$), 2.02 (dt, 2H, $C_5\underline{H}$). ^{13}C-NMR (126 MHz, $CDCl_3$, ppm) δ: 171.56 ($\underline{C_4}$), 169.79 ($\underline{C_7}$), 151.11 ($\underline{C}_{Ar}$), 148.07 ($\underline{C}_{Ar}$), 132.16 ($\underline{C}_{Ar}$), 129.09 ($\underline{C}_{Ar}$), 128.58 ($\underline{C}_{Ar}$), 128.29 ($\underline{C}_{Ar}$), 128.23 ($\underline{C}_{Ar}$), 127.73 ($\underline{C}_{Ar}$), 127.18 ($\underline{C}_{Ar}$), 125.53 ($\underline{C}_{Ar}$), 123.07 ($\underline{C}_{Ar}$), 122.55 ($\underline{C}_{Ar}$), 114.80 ($\underline{C_1}$), 107.74 ($\underline{C_2}$), 70.86 (-$\underline{C}H_2$-$\underline{C}H_2$-O-), 70.56 (-$\underline{C}H_2$-$\underline{C}H_2$-O- *and* $\underline{C_8}$), 70.27 (-$\underline{C}H_2$-$\underline{C}H_2$-O-), 55.45 ($\underline{C_3}$), 34.78 ($\underline{C_5}$, $\underline{C_6}$). MALDI-TOF: *m/z* = 1378, 1422, 1466, 1510, 1554, 1598, 1642, 1686, 1730, 1774, 1818, 1862 and 1906 which corresponds to DBCO-$(CH_2CH_2O)_n$-DBCO+Na^+ (n= 14 - 26) *and* 1570, 1614, 1658 *and* 1702 which corresponds to DBCO-$(CH_2CH_2O)_n$-DBCO+K^+ (n= 17 – 20). FT-IR ν_{max} film (cm^{-1}): 3507 (N-H), 2870 (C-H), 1655 (C=O amide), 1535, 1466, 1449, 1398, 1325, 1250, 1206, 1101 (C-N), 949, 846, 729. UV-Vis λ_{max} (25 °C, milliQ) = 290.5 nm. $\varepsilon_{290.5}$ (milliQ) = 16430 L mol^{-1} cm^{-1}.

Small angle scattering experiments

Setup. All SAXS measurements were performed analogous to earlier work.[3] SAXS measurements were carried out at the BM26B station at the European Synchrotron Radiation Facility (ESRF) in Grenoble, France. X-ray radiation with a wavelength of λ = 0.1 nm. The sample-to-detector distance was 2.7 m. The SAXS images were recorded on a noiseless, solid-state, highly sensitive Pilatus 1M 3S detector with a pixel size of 172 X 172 *μ*m and an array dimension of 981 X 1043 pixels. The scattering angle 2θ and beam center scale were calibrated using the diffraction peak positions of a silver behenate standard powder sample. The SAXS images were normalized by the primary beam intensity fluctuation. The background scattering was scaled for the sample transmission prior to perform the background subtraction. Background-subtracted images were radially integrated around the beam center using a python suite to obtain the $I(q)$ versus q profiles, where $q = 4\pi/\lambda \sin\theta$. Samples were measured in 2 mm quartz capillaries and were inserted in a Linkam hot stage for temperature control in the range of 5 to 50 °C. Absolute intensities were obtained using the secondary standard method using pure water.[4]

Fitting procedures. The SAXS curves were fitted using the SASfit software.[5] The scattering of polymers in solution could be described using a wormlike chain model described by Kholodenko:[6]

$$I(q) = (\Delta\rho)^2 \varphi P_0(q, L, 2l_p) P_{CS}(q, R)$$

where $\Delta\rho = \rho_{\text{polymer}} - \rho_{\text{water}}$ is the difference in electron density between the polymer chain and the solution, φ is the polymer volume fraction, L is the contour length, l_p is the persistence length and R is the cross-sectional radius of the polymer chain.

The scattering from the network architecture was described using a combination of the wormlike model[6] and of the model developed by Ornstein and Zernike,[7] which describes the scattering from network heterogeneities, according to the equation:

$$I(q) = I_{\text{bundles}}(q) + I_{\text{network}}(q) = (\Delta\rho)^2 \varphi P_0(q, L_B, 2l_{p,B}) P_{CS}(q, R_B) + \frac{I(0)}{1 + q^2 \xi_{OZ}^2}$$

Where L_B is the polymer contour length, $l_{p,B}$ is the persistence length of the polymer bundle, R_B is the cross-sectional radius of the bundles, $I(0)$ is the forward scattering of the OZ-term, and ξ_{OZ} is the correlation length of the network heterogeneities. Because L of the bundles is larger than the SAXS resolution, we kept L_B fixed at 930 nm. Additionally, to reduce the amount of fitting parameters and considering that the persistence length of the bundles is relatively large and does not have a major effect on the model in the fitted q range, we fixed $l_{p,B}$ to 273 nm for all gels. Finally, since the correlation length of the network heterogeneities is larger than the SAXS resolution ($\xi_{OZ} > 80$ nm), we kept this parameter fixed at 100 nm for all gels. The individual contributions of the models and R_B were fitted. The error of the bundle size R_B was determined after fitting the scattering curves, by changing the value until the fit did not converge anymore.

For the crosslinked gels at low T, a second contribution of the wormlike model[6] was added to account for the re-dissolved polymer ends and small polymers:

$$I(q) = I_{\text{bundles}}(q) + I_{\text{network}}(q) + I_{\text{polymers}}(q)$$

$$= (\Delta\rho)^2 \varphi P_0(q, L_B, 2l_{p,B}) P_{CS}(q, R_B) + \frac{I(0)}{1 + q^2 \xi_{OZ}^2} + (\Delta\rho)^2 \varphi P_0(q, L, 2l_p) P_{CS}(q, R)$$

Again, $L_{p,B}$, $l_{p,B}$ and ξ_{OZ} were fixed at abovementioned values. Additionally, we assume that for the re-dissolved polymer-ends and small polymers, the polymer cross-sectional radius R and persistence length l_p is the same for all samples at $T = 5$ °C. These parameters were therefore obtained from the scattering curve of the non-crosslinked gel **1** at 5 °C. The individual contributions of the models and R_B were fitted. The error of the bundle size R_B was determined after fitting the scattering curves, by changing the value until the fit did not converge anymore. All fitting results are shown in Supplementary Table 2.

UV-Vis experiments

All UV-Vis measurements were carried out on a *JASCO V-630 UV-Vis spectrophotometer* using a 2 mm path length HELLMA quartz cuvette. For the conversion measurements a spectrum with the range of $\lambda = 350 - 250$ nm was obtained every 15 seconds. After background subtraction the conversion of DBCO was calculated using the extinction coefficient at $\lambda = 304$ nm ($\varepsilon_{304} = 7610$ L mol^{-1} cm^{-1}). Note that a large background correction was necessary due to background absorption and baseline spreading caused by uncontrolled heating. These corrections result in an error of ≥ 10 % in all UV-Vis measurements. The concentrations of **2a** used for the UV-Vis and rheology measurements as given in Figure 4a of the main manuscript are summarized in Supplementary Table 1. The concentration of **1** was 1.0 mg mL^{-1} ($[N_3] = 104.1$ µM) for all measurements.

Supplementary Table 1 | Crosslinker concentrations and absolute moduli. Concentrations of crosslinker **2a** used for the UV-vis and rheology measurements to study the dependence on crosslinker density and the storage moduli *G′* of the corresponding crosslinked gels at *T* = 37 °C (crosslink temperature) and *T* = 5 °C.

**ratio [DBCO]/[N$_3$]**	**[2a] (µM)**	***G′* at *T* = 37 °C (Pa)**	***G′* at *T* = 5 °C (Pa)**
0	0	136	<1
0.1	5.2	157	7
0.2	10.4	140	20
0.5	26.1	211	78
0.9	46.8	148	72
1	52.1	201	93
1.25	65.1	184	95
1.5	78.2	125	48
1.75	91.0	115	22
2.0	104.1	125	20

Supplementary Table 2 | Small angle X-ray scattering fitting data. Samples: **1**+**2a** gel, crosslinked at T_{cl} = 25, 30, 40 and 50 °C; **1**+**2b** gel, crosslinked at T_{cl} = 37 °C; and the non-crosslinked blank **1** (all with PIC concentrations c_{PIC} = 4 mg mL^{-1}). Data is available at the crosslinking temperature, after the first cooling to 5 °C, after heating to 50 °C and after the second cooling to 5 °C where all samples show comparable results.

Sample	T_{cl} (°C)	$T = T_{cl}$				T = 5 °C				T = 50 °C				T = 5 °C			
		Bundle		Polymer	Network	Bundle		Polymer	Network	Bundle		Polymer	Network	Bundle		Polymer	Network
		$(\Delta\rho)^2\varphi$	R_B (nm)	$(\Delta\rho)^2\varphi$	I_0	$(\Delta\rho)^2\varphi$	R_B (nm)	$(\Delta\rho)^2\varphi$	I_0	$(\Delta\rho)^2\varphi$	R_B (nm)	$(\Delta\rho)^2\varphi$	I_0	$(\Delta\rho)^2\varphi$	R_B (nm)	$(\Delta\rho)^2\varphi$	I_0
**1+2a**	25	0.1	1.5	0.04	1.3	0.09	7.2	0.07	0.3	0.3	3.1	—	1.9	0.2	7.9	0.06	0.15
**1+2a**	30	0.1	3.2	0.04	1.8	0.2	7.4	0.06	0.8	0.4	3	—	1.6	0.3	7.3	0.06	0.9
**1+2a**	40	0.5	3.4	—	2	0.9	7.4	0.04	0.8	0.7	4	—	1.7	1.1	7.3	0.03	1.2
**1+2a**	50	0.7	4.3	—	2.3	0.9	7.5	0.04	0.9	0.7	4.3	—	2.3	1.1	7.4	0.05	0.6
**1+2b**	37	0.2	2.2	—	2.7	0.7	6.2	0.04	1.8	0.6	3.4	—	2	1.2	5.4	0.06	1.1
**1**	—					—	—	0.06	—	0.05	2.6	—	1.7				

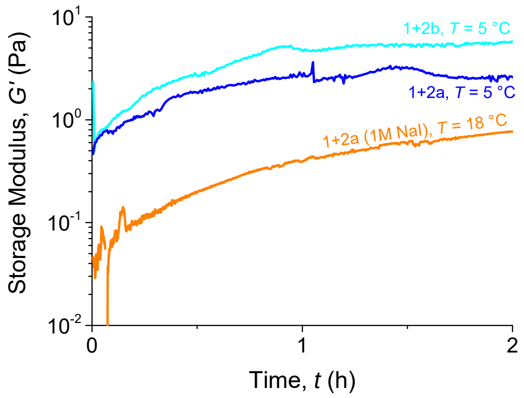

Supplementary Figure 1 | Crosslinking below T_{gel}. Storage modulus G' of PIC solutions **1+2a** (blue) and **1+2b** (light blue), crosslinked at T_{cl} = 5 °C, and of PIC solution **1+2a** with 1M NaI at T_{cl} = 18 °C. For all experiments, [**1**] = 1 mg mL^{-1}, equivalent to 104 μM azide groups, [**2a**] = [N_3]/2 = 52 μM.

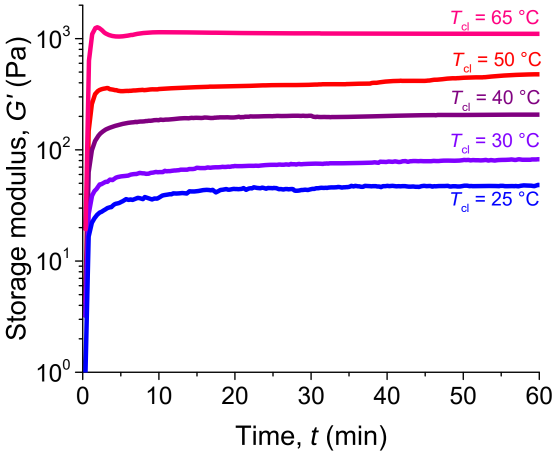

Supplementary Figure 2 | Mechanical properties during the crosslinking reaction at all different crosslinking temperatures. Storage moduli of mixtures of PIC hydrogels and **2a** crosslinked between T_{cl} = 25–65 °C. In all experiments, [**1**] = 1 mg mL^{-1}, equivalent to 104 μM azide groups, [**2a**] = [N_3]/2 = 52 μM. The samples were mixed below 5 °C, applied to the rheometer and heated immediately to the crosslink temperature and the storage modulus (at γ = 0.04, f = 1 Hz) was followed in time.

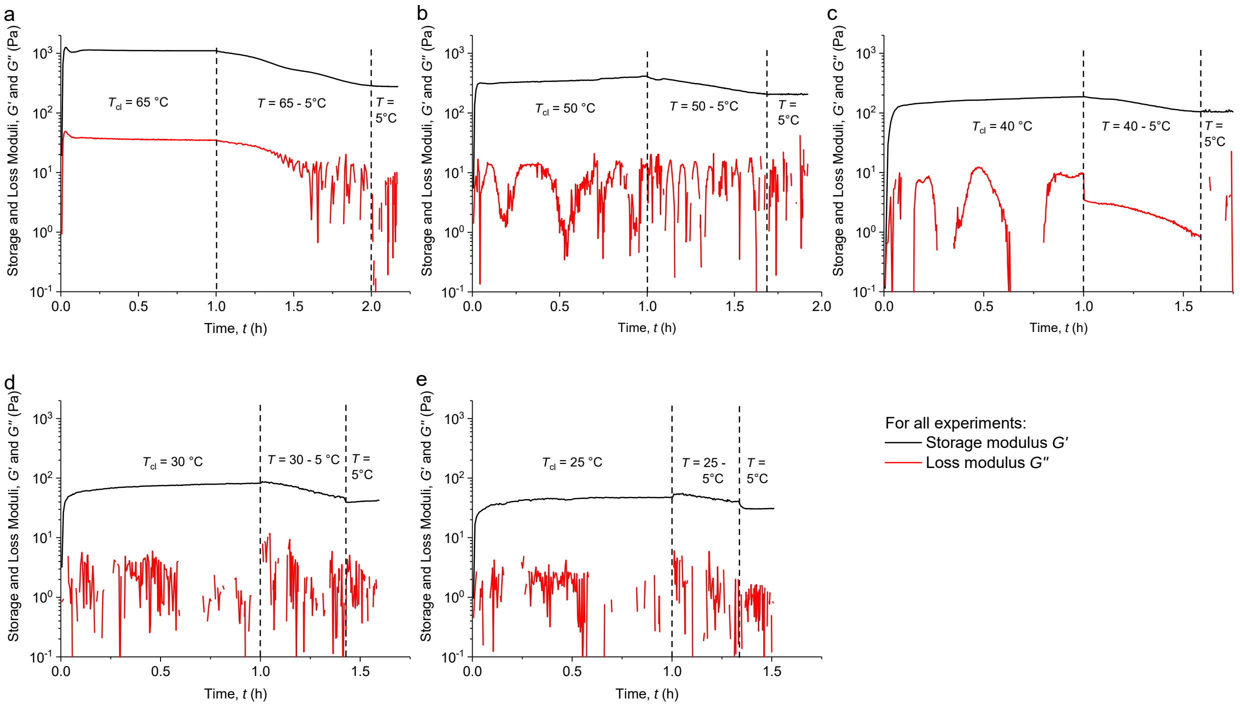

Supplementary Figure 3 | Mechanical properties during the crosslinking reaction at all different crosslinking temperatures and after cooling. Storage (black data) and loss (red data) moduli of mixtures of PIC hydrogels and **2a** crosslinked between T_{cl} = 25–65 °C (**a**-**e**). In all experiments, [**1**] = 1 mg mL^{-1}, equivalent to 104 μM azide groups, [**2a**] = [N_3]/2 = 52 μM. The samples were mixed below 5 °C, applied to the rheometer and heated immediately to the crosslink temperature and held for 1 hr. Then the sample was cooled to 5 °C (cooling rate 1 °C min^{-1}) and held for 10 min. During the experiment, the moduli (at γ = 0.04, f = 1 Hz) were followed in time. Note that the loss contribution to the stiffness of the PIC networks is small and, particularly at low temperatures, very noisy.

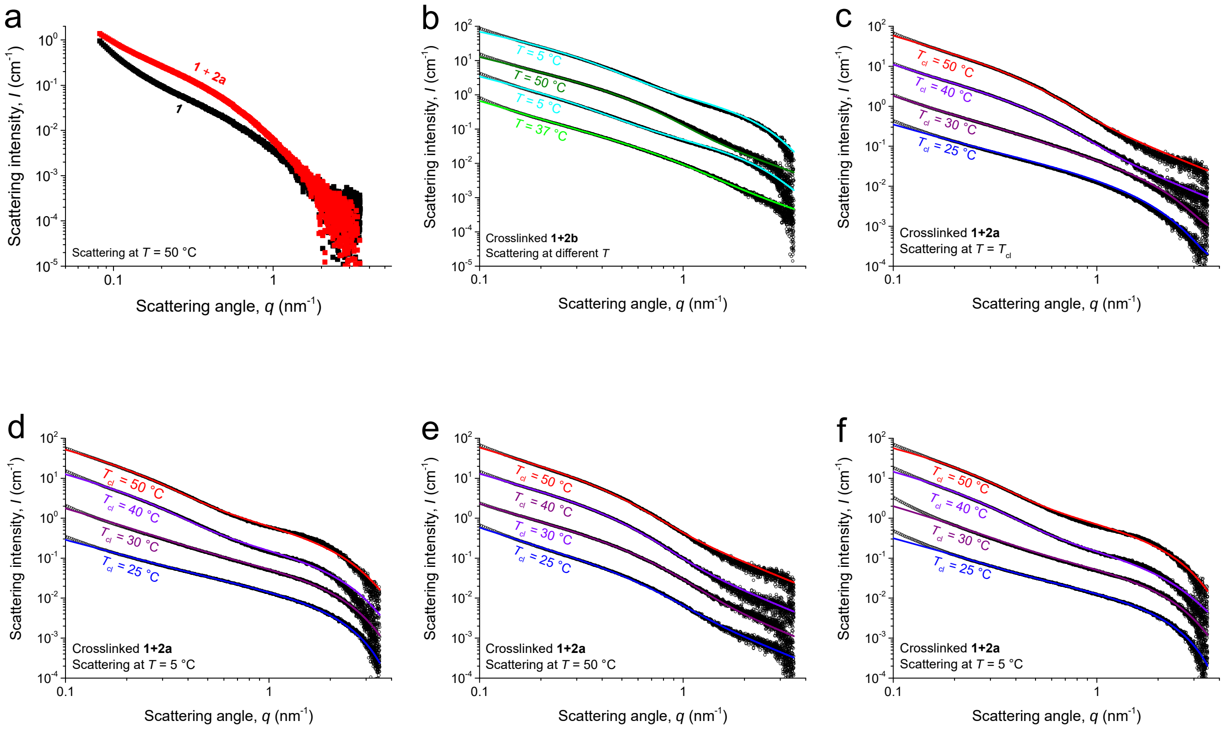

Supplementary Figure 4 | Shifted scattering curves and fits for crosslinked gels 1+2a or 1+2b. (**a**) Scattering patterns of a non-crosslinked PIC gel **1** and a crosslinked PIC gel **1+2a**, measured at T = 50 °C. (b) SAXS curves of (**b**) **1+2b** gel, crosslinked at 37 °C, at the crosslinking temperature (green), 5 °C (blue) and 50 °C (dark green), and of (**c**) **1+2a** gels crosslinked at 25, 30, 40 and 50 °C at the crosslinking temperatures (**c**) and after cooling to 5 °C (**d**, **f**), and after heating to 50 °C (**e**) with the best fit to the model (solid lines). The curves were shifted vertically to improve visibility. Note that the fitting results are summarized in Supplementary Table 2.

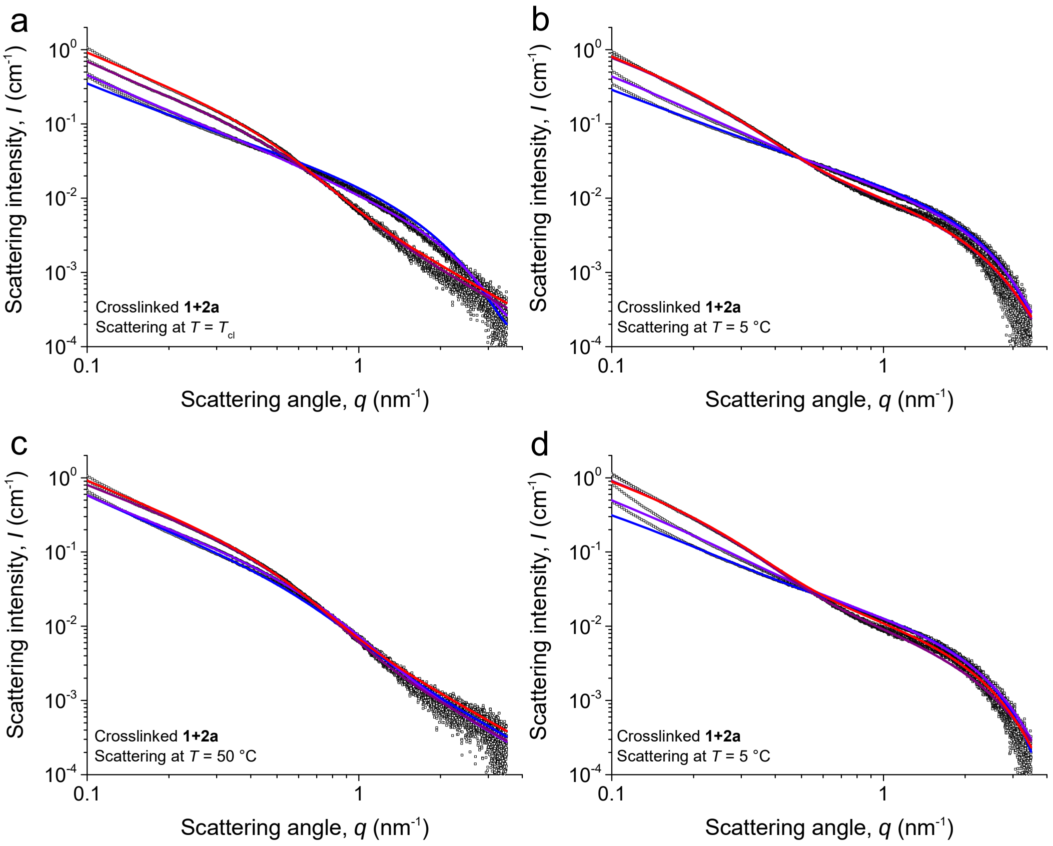

Supplementary Figure 5 | Un-shifted scattering curves and fits for crosslinked gels 1+2a. (**a**) **1+2a** gels crosslinked at 25 (blue), 30 (violet), 40 (purple) and 50 °C (red) at the crosslinking temperatures (**a**) and after cooling to 5 °C (**b**, **d**), and after heating to 50 °C (**c**) with the best fit to the model (solid lines). Note that the fitting results are summarized in Supplementary Table 2.

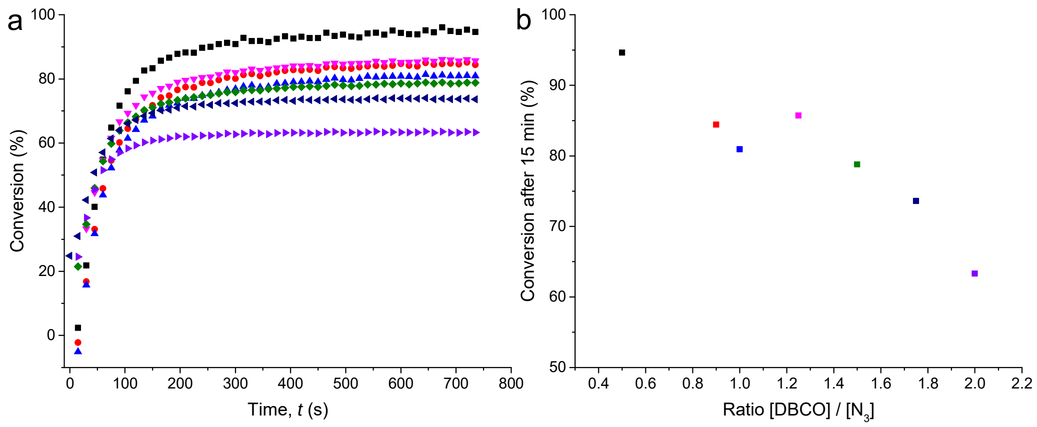

Supplementary Figure 6 | Conversion of the crosslinking reaction. (**a**) UV-vis analysis of the conversion of DBCO in the PIC hydrogels using an increasing concentration of **2a**. (**b**) Calculated conversion of DBCO after 15 minutes plotted against the ratio [DBCO]/[N_3], See supplementary Table 1.

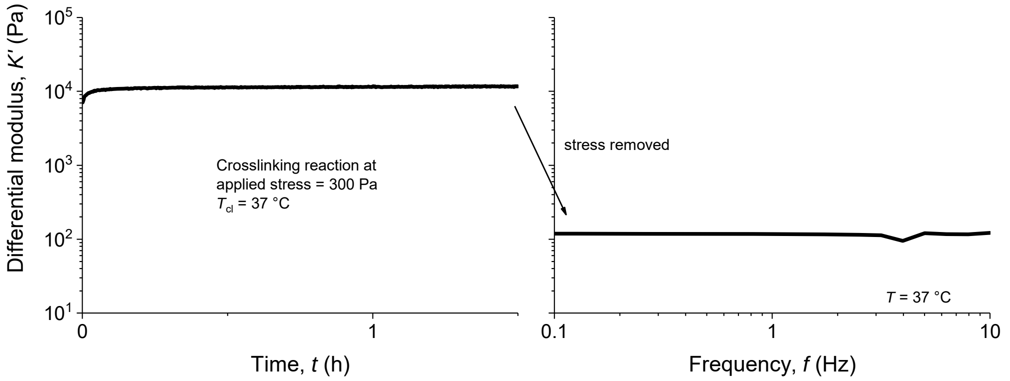

Supplementary Figure 7 | Crosslinking under stress. Differential modulus *K*' of a PIC solution **1+2a**, crosslinked for 90 minutes at T_{cl} = 37 °C with an applied pre-stress of σ = 300 Pa (with $\delta\sigma$ = 3 Pa and f = 1 Hz) and after removal of the pre-stress σ = 0 Pa (γ = 0.04 and f = 1 Hz). For the experiment, [**1**] = 1 mg mL^{-1}, equivalent to 104 μM azide groups, [**2a**] = $[N_3]/2$ = 52 μM. The sample was mixed below 5 °C, applied to the rheometer and heated immediately to 37 °C (30 s) to allow gel formation and the stress was applied. After 90 min, the stress was removed and the mechanical properties were evaluated with a frequency sweep.

Supplementary References

1 van Beijnen, A. J. M., Nolte, R. J. M., Drenth, W., Hezemans, A. M. F. & van de Coolwijk, P. J. F. M. Helical Configuration of Poly(iminomethylenes). Screw Sense of Polymers Derived from Optically Active Alkyl Isocyanides. *Macromolecules* **13** (1980) 1386.

2 Mandal, S. *et al.* Therapeutic nanoworms: towards novel synthetic dendritic cells for immunotherapy. *Chem. Sci.* **4** (2013) 4168.

3 Jaspers, M., Pape, A. C. H., Voets, I. K., Rowan, A. E., Portale, G. & Kouwer, P. H. J. Bundle Formation in Biomimetic Hydrogels. *Biomacromolecules* **17** (2016) 2642.

4 Orthaber, D., Bergmann, A. & Glatter, O. SAXS experiments on absolute scale with Kratky systems using water as a secondary standard. *J. Appl. Crystallogr.* **33** (2000) 218.

5 Kohlbrecher, J. SASfit: A Program for Fitting Simple Structural Models to Small Angle Scattering Data. *Paul Scherrer Institut, Laboratory for Neutron Scattering* (2008)

6 Kholodenko, A. L. Analytical calculation of the scattering function for polymers of arbitrary flexibility using the dirac propagator. *Macromolecules* **26** (1993) 4179.

7 Ornstein, L. S. & Zernike, F. *KNAW Proc.* (1914) 793.
